# The management of Castleman's disease of the mediastinum: a case report

**DOI:** 10.1186/1757-1626-1-330

**Published:** 2008-11-19

**Authors:** Panagiotis Hountis, Panagiotis Dedeilias, Mattheos Douzinas

**Affiliations:** 1 Thoracic Surgery Department, Athens Naval Hospital, Deinokratous 70 Athens, Greece; 2 Cardiac Surgery Department, Evaggelismos General Hospital Ipsilantou 45-47 Athens, Greece

## Abstract

Castleman's disease or angiofollicular lymph node hyperplasia is a rare clinical entity that may present in many sites and with a variety of symptoms. We report here a case of unicentric Castleman's disease (hyaline vascular pattern) located in the mediastinum as a solitary mass. The patient was a Caucasian female 58 years old presented after incidental discovery of the mass in a x-ray. In Castleman's disease surgery is generally recommended for localised lesions to remove the mass as completely as possible reserving other treatment modalities for unresectable cases or multicentric disease.

## Background

Castleman's disease (CD) is a rare atypical lymphoproliferative disorder often also called angiofollicular lymph node hyperplasia. It was first described as a clinical entity in 1956. [1] May present in many sites and a variety of symptoms. The disease has been subdivided in two patterns with intermediate variants. 1. unicentric pattern (or hyaline vascular) and 2. multicentric pattern (plasma cell pattern). There are reports in the literature of mixed patterns as well. The unicentric pattern occurs much more frequently than the multicentric and is usually localized to the mediastinum or pulmonary hilum. The multicentric involves lymph nodes separately or in aggregations and often displays multicentricity with systemic symptoms including autoimmune phenomena and aggressive course. Infections are the most frequent cause of death in these cases followed by malignancies such as Kaposi sarcoma, malignant lymphoma and epithelial neoplasia. Other complications present may be pleural effusions and substernal goiter. The differential diagnosis should include diseases that present with large mediastinal masses, such as Hodgkin's lymphomas and thymomas, as well as certain rheumatologic diseases that may cause lymph node enlargement.[2]

## Case presentation

**Figure 1:**
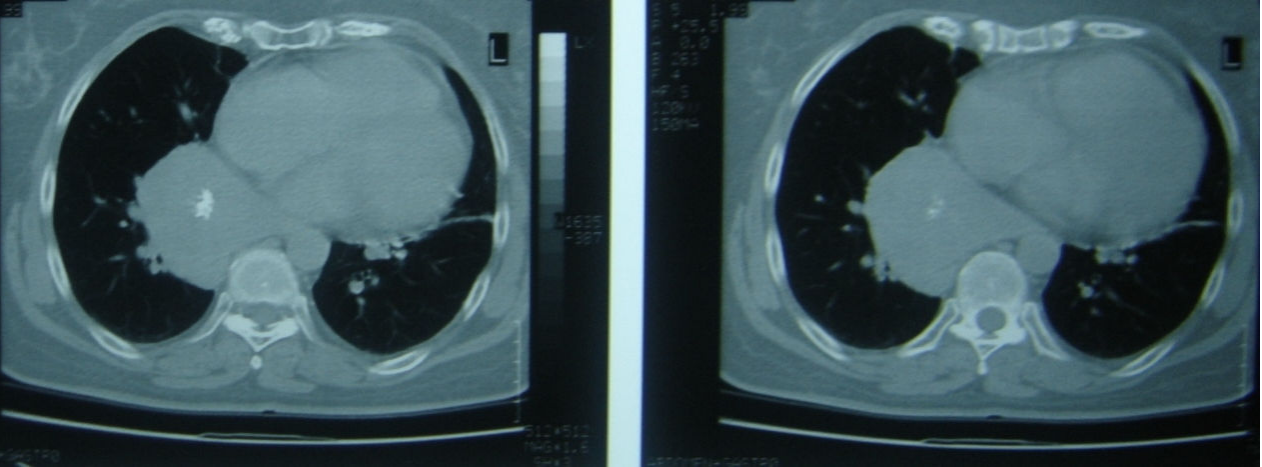
Computerized tomography of the patient showing the right lower posterior mediastinal mass.

The case was a 58 year old Caucasian Greek female (weight 78 kgr, height 162 cm) without a significant past occupational, medical and family history. She had 2 vaginal deliveries 30 and 28 years before. The patient had no history of smoke or alcohol use. The patient was presented with an asymptomatic mass in a chest x ray that was performed for a routine check up. A CT scan (Figure 1) confirmed the presence of a mass at the right posterior mediastinum that was not accessible for FNA (fine needle aspiration) biopsy. She had no fever or secondary symptoms of malignancy. Abdominal and brain CT (Computerized Tomography) scan was negative. Barium swallow examination of the esophagus was negative for pathology. The patient was subjected to right posterolateral thoracotomy with radical excision of the mass. The lung was easily freed and the mass was completely excised encapsulated in a clear 'plan de cleavage'

Pathology examination of the specimen showed Castleman's disease.(hyaline vascular pattern). According to the patterns of the disease there was no need for additional treatment. The hyaline vascular subtype was characterized by multiple germinal centers surrounded by circumferential arranged layers (onion skin) of small lymphocytes interconnected by a prominent vascular stroma with occasional plasma cells.

Follow up of the patient for the last 8 years has showed that she is free of the disease.

## Discussion

Castleman's disease is an eponymous which was first given to a lymphoid tumour of the mediastinum [3] also referred as lymph node hyperplasia, or angiofollicular lymph node hyperplasia. Although the majority of lesions occur within the chest, less commonly other sites including neck, pelvis, retroperitoneum and axilla may be involved.[4]

The frequent concomitance of hyaline vascular pattern and plasma cell patterns at separate sites together with transient morphological patterns from one type to the other and from localized to multicentric form during the course of the disease have suggested that Castleman's disease is a single disorder related to immune dysregulation. This is supported by the B and T cell impaired function and the frequent development of autoantibodies. A key event in the pathogenesis of Castleman's disease has been recently suggested to be and abnormal production of a B cell growth factor such as IL-6 (Interleukin 6), leading to lymphoproliferation and plasma cell differentiation and being involved in the pathogenesis of plasmacytomas. In this event, Kaposi sarcoma associated virus (Human Herpes Virus-8) which has been found in many cases of CD especially in the multicentric form could play a crucial role in producing IL-6 and releasing angiogenic factors. It has been already discussed that multicentric presentation is associated with poor prognosis and the development of lymphoma and infections.

## Conclusion

Mediastinal localization is a rare manifestation of Castleman's disease often diagnosed after onset of non-specific thoracic symptoms such as dyspnoea, cough, chest-wall pain or generalized malaise; occasionally patients could be asymptomatic.

Tissue diagnosis is mandatory in all cases to avoid mismanagement. It is important to know that needle biopsy has low diagnostic accuracy and thoracoscopic biopsy is dangerous because of the high vascularization of the tumour increasing risk of bleeding.

Complete surgical removal is usually curative alone. Radical excision is mandatory but often the tumour is not easily removed from the underlying tissues. It should be stressed that although adjuvant chemotherapy has been used in these cases subtotal excision has been performed without short-term recurrences reported. [5]

## Consent

Written informed consent was obtained from the patients for publication of this case report and accompanying images. A copy of the written consent is available for review by the Editor-in-Chief of this journal.

## Competing interests

The authors declare that they have no competing interests.

## Authors' contributions

PH analyzed and interpreted the patient data and wrote the manuscript. PD was a major contributor in writing the manuscript. MD cared for the patients and operated them. All authors read and approved the final manuscript.
